# Internalization of novel non-viral vector TAT-streptavidin into human cells

**DOI:** 10.1186/1472-6750-7-1

**Published:** 2007-01-02

**Authors:** Johanna Rinne, Brian Albarran, Juulia Jylhävä, Teemu O Ihalainen, Pasi Kankaanpää, Vesa P Hytönen, Patrick S Stayton, Markku S Kulomaa, Maija Vihinen-Ranta

**Affiliations:** 1 NanoScience Center; Department of Biological and Environmental Science, University of Jyväskylä, Jyväskylä, Finland; 2 Department of Bioengineering, University of Washington, Seattle, WA, USA; 3 Department of Materials, ETH Zürich, Zürich, Switzerland; 4 Institute of Medical Technology, University of Tampere, Tampere, Finland

## Abstract

**Background:**

The cell-penetrating peptide derived from the Human immunodeficiency virus-1 transactivator protein Tat possesses the capacity to promote the effective uptake of various cargo molecules across the plasma membrane *in vitro *and *in vivo*. The objective of this study was to characterize the uptake and delivery mechanisms of a novel streptavidin fusion construct, TAT_47–57_-streptavidin (TAT-SA, 60 kD). SA represents a potentially useful TAT-fusion partner due to its ability to perform as a versatile intracellular delivery vector for a wide array of biotinylated molecules or cargoes.

**Results:**

By confocal and immunoelectron microscopy the majority of internalized TAT-SA was shown to accumulate in perinuclear vesicles in both cancer and non-cancer cell lines. The uptake studies in living cells with various fluorescent endocytic markers and inhibiting agents suggested that TAT-SA is internalized into cells efficiently, using both clathrin-mediated endocytosis and lipid-raft-mediated macropinocytosis. When endosomal release of TAT-SA was enhanced through the incorporation of a biotinylated, pH-responsive polymer poly(propylacrylic acid) (PPAA), nuclear localization of TAT-SA and TAT-SA bound to biotin was markedly improved. Additionally, no significant cytotoxicity was detected in the TAT-SA constructs.

**Conclusion:**

This study demonstrates that TAT-SA-PPAA is a potential non-viral vector to be utilized in protein therapeutics to deliver biotinylated molecules both into cytoplasm and nucleus of human cells.

## Background

Due to the limitations of current drug delivery systems, which have been hampered by their inefficiencies in traversing the cell membrane, there is a pressing need to develop methods for increasing intracellular delivery of protein-based cargoes. Over the past decade, numerous strategies to overcome the cell membrane barrier have been proposed, including electroporation, microinjection, viral vectors, liposome encapsulation and receptor-mediated endocytosis. These methods have, however, been plagued by low delivery efficiencies and, to some extent, increased cellular toxicity. Naturally occurring short cell-penetrating peptides (CPPs) derived from viral, insect or mammalian proteins, have attracted considerable interest in the field of drug delivery for their ability to direct cellular uptake through active transport mechanisms. CPPs are oligopeptides, 11–30 amino acid residues in length, that are capable of conferring their apparent translocation activity to proteins and other macromolecular cargo to which they are linked [1]. In recent years, CPPs have been studied extensively, both *in vitro *and *in vivo*, for their ability to delivery an array of pharmalogically relevant cargoes, such as antisense oligonucleotides, peptides, proteins, plasmids, liposomes and nanometer-sized particles, with encouraging results [2]. Lately, CPPs have also been used to treat preclinical models of human disease [3,4].

One of the most well-studied and efficient cell penetrating peptides is the 11-amino-acid peptide of the Human immunodeficiency virus type 1 (HIV-1) Tat protein. This basic region of Tat, containing amino acid residues 47–57 (YGRKKRRQRRR; TAT_47–57_) [1,5], is crucial for many key functions of the protein, including interaction with the transactivation-responsive region in viral mRNA [6], nuclear localization [7]and most importantly, cellular uptake [8,9]. TAT_47–57 _has been shown to direct the internalization of an extensive list of cargoes ranging from small peptides [4] to proteins and polymers [3,10-12], liposomes [13,14], phage vectors [15], plasmid DNAs [16,17] and even nanoparticles [18]. Moreover, TAT_47–57 _has also been used *in vivo *to deliver biologically active β-galactosidase into all tissues of the mouse, even the brain [12].

A variety of internalization routes for the TAT_47–57 _sequence and TAT-mediated cargoes have been suggested. In the study of Fittipaldi *et al*. (2003) and Ferrari *et al*. (2003) Tat11EGFP and GST-Tat-eGFP proteins were reported to internalize into cells via caveolae-mediated endocytosis and transported further to the perinuclear area via an actin cytoskeleton-mediated mechanism [19,20]. Wadia *et al*. (2004) in turn showed the internalization of the TAT-Cre protein into cells by lipid raft-dependent macropinocytosis [21], and Richard *et al*. (2005) suggested the uptake of the TAT peptide via clathrin-mediated endocytosis [22]. Recently, also Säälik *et al*. (2004) demonstrated the uptake of biotinylated TAT, detected with FITC-labeled avidin, via both clathrin-dependent and clathrin-independent endocytosis [23]. Central to the use of TAT, however, is not only its ability to deliver cargo to cells but, importantly, its non-cytotoxicity and stable biological activity over long time periods [5].

Core streptavidin (SA; 125–127 aa) from *Streptomyces avidinii *has been used in many pharmalogical applications. The exact mechanism of its cellular uptake and intracellular delivery is, however, not well known. The internalization of SA has been suggested to occur via receptor-mediated endocytosis, involving lysine residues [24] and the RYD sequence (Arg-Tyr-Asp) [25]. *In vivo*, the biodistribution of SA has been shown to exhibit slow clearance from the bloodstream due to accumulation in the kidney [26,27]. The present study was designed to gain insight into the internalization of a novel TAT-streptavidin (TAT-SA) construct [10] in human cells. Additionally, the ability of TAT-SA as a transporter of biotin and biotinylated molecules was examined. The subcellular distribution of TAT-SA was altered by biotinylated, pH-responsive polymer poly(propylacrylic acid) (PPAA), which further promoted the endosomal release. These studies provide insights into the mechanism of TAT-SA uptake in cells and may have implications for the optimal use of TAT-SA and PPAA for the intracellular delivery of numerous biotinylated macromolecules.

## Results

### Characterization of TAT-SA constructs

The structure of TAT-streptavidin (TAT-SA) has been previously described [10] as a tetrameric fusion protein, in which the TAT_47–57 _peptide has been attached to the N-terminus of each streptavidin monomer. Biotins or biotinylated molecules are located into binding pockets of SA. In this study, the stability and biotin-binding ability of TAT-SA were analyzed by sodium dodecyl sulphate-polyacrylamide gel electrophoresis (SDS-PAGE) and immunoblot analysis. Both constructs, TAT-SA and Alexa488-labeled TAT-SA (TAT-SA-A488), retained their tetrameric (60 kD) conformations in reducing conditions as either apoform or bound to biotin. When samples were preheated to 68°C, a minor proportion of TAT-SA or TAT-SA-A488 were detected as monomeric (15 kD) or dimeric (30 kD) forms. Binding of biotin to TAT-SA, however, stabilized the constructs at 68°C, since only tetrameric proteins were detected (unpublished data). Importantly, reducing conditions and preheatmeant to 37°C did not change the tetrameric conformations of either TAT-SA or TAT-SA bound to biotin (Additional file 1).

### Microscopical analysis of TAT-SA and SA uptake

**Figure 1:**
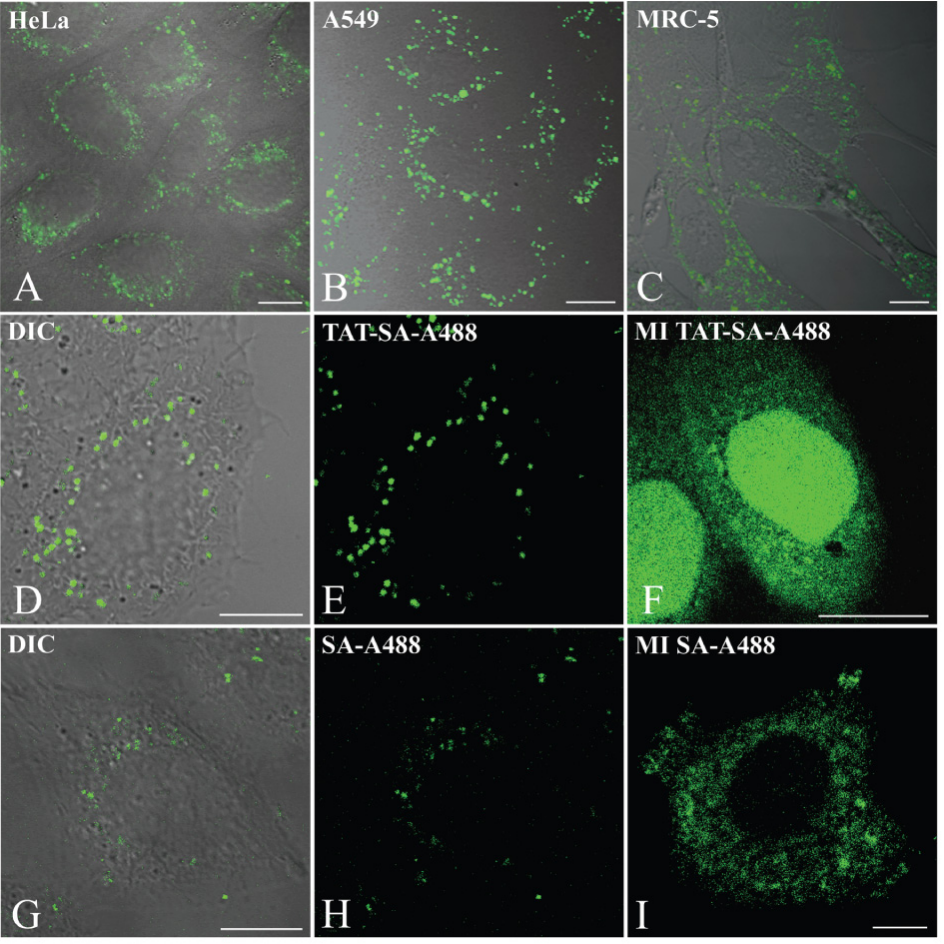
**Confocal microscopy analysis of intracellular distribution of TAT-SA or SA at 4 h post transduction or injection**. (A-C) Human cancer cell lines (HeLa, A549) and a non-cancer cell line (MRC-5) were transduced with TAT-SA prior to PFA fixation, permeabilization with Triton-X and immunolabeling with rabbit SA Ab followed by Alexa-488-conjugated goat anti-rabbit IgG (green). (D-E) Living HeLa cells were transduced or (F) injected to the cytoplasm with TAT-SA-A488 (green), or (G-H) transduced or (I) injected with SA-A488 (green). Scale bars, 10 μm.

TAT-SA (2 μM) was shown to internalize into human epithelial carcinoma (HeLa), human lung carcinoma (A549) and human lung fibroblast (MRC-5) cell lines at 4 h post transduction (Fig. 1A–C). In living HeLa cells TAT-SA-A488 was observed to cross the cell membrane rapidly, starting at 5 min post transduction, by confocal microscopy. At later timepoints the majority of TAT-SA-A488 was localized in vesicular compartments within the cytoplasm, and only few cells displayed detectable nuclear accumulation at 4 h post transduction (Fig. 1D–E). Control studies in which cells were first transduced either with TAT-SA-A488 or TAT-SA, fixed at 4 post transduction and immunostained for SA confirmed that both studies in living and fixed cells resulted in a similar subcellular localization. Additionally, in cells transduced with SA-A488 (2 μM) alone, the cellular uptake was reduced (Fig. 1G–H). The role of TAT during the nuclear import of SA was confirmed by injecting TAT-SA-A488 or SA-A488 (~1.5 × 10^6 ^molecules/cell) directly into the cytosol of HeLa cells. At 4 h post injection the majority of TAT-SA-A488 was found to accumulate in the nucleoplasm with only minor localization in the cytoplasm. In the nucleus TAT-SA-A488 was distributed evenly throughout the nucleoplasm without accumulation into distinct subnuclear structures (Fig. 1F). In contrast, SA-A488 was distributed randomly throughout the cytoplasm but not in the nucleus (Fig. 1I).

**Figure 2:**
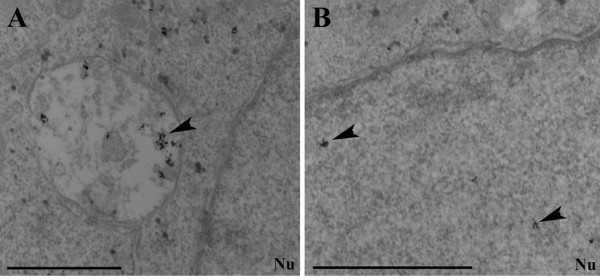
**Localization of internalized TAT-SA in HeLa cells by immunoelectron microscopy at 4 h post transduction**. Intracellular TAT-SA was detected with pre-embedding immunolabeling technique, in which anti-SA staining was followed by silver-enhanced nanogold and gold-toning treatments. Localization of SA was shown as dark spots (arrow heads) within (A) cytoplasmic vesicular structures and further (B) in the nucleus of cells (nu). Scale bars, 1 μm.

To monitor further the internalization and intracellular transport of TAT-SA and SA, transduced HeLa cells were labeled with an antibody against SA and nanogold-immunolabeling electron microscopy with a silver enhancement technique was used. Intracellular TAT-SA, visualized as small, intensely labeled, grainy spots was localized in large intracellular vesicles near the cell surface or in endocytic-like vesicles adjacent to the nuclear membrane at 4 h p.t (Fig. 2A). At the same time a small proportion of TAT-SA was observed in the cytosol and the nucleus of cells (Fig. 2B). Interestingly, in the control experiments SA was observed in smaller cytoplasmic vesicles without any nuclear localization than in those displaying TAT-SA at 30 min to 4 h post transduction.

### Endocytic entry pathways of TAT-SA

To study the potential role of caveolar endocytosis in the uptake of TAT-SA, HeLa cells were transduced and immunolabeled with caveolin-1 antibody. No colocalization of TAT-SA-A488 and caveolin was, however, apparent at 15–60 min post transduction. In addition, the internalization of TAT-SA-A488 into living HeLa cells was not blocked in the presence of the cholesterol-depleting agent β-methylcyclodextrin, a known inhibitor of caveolae-mediated endocytosis. Importantly, control studies with human hepatoma (HepG2) cells, which do not express caveolins endogenously, demonstrated the efficient internalization of TAT-SA (unpublished data).

**Figure 3:**
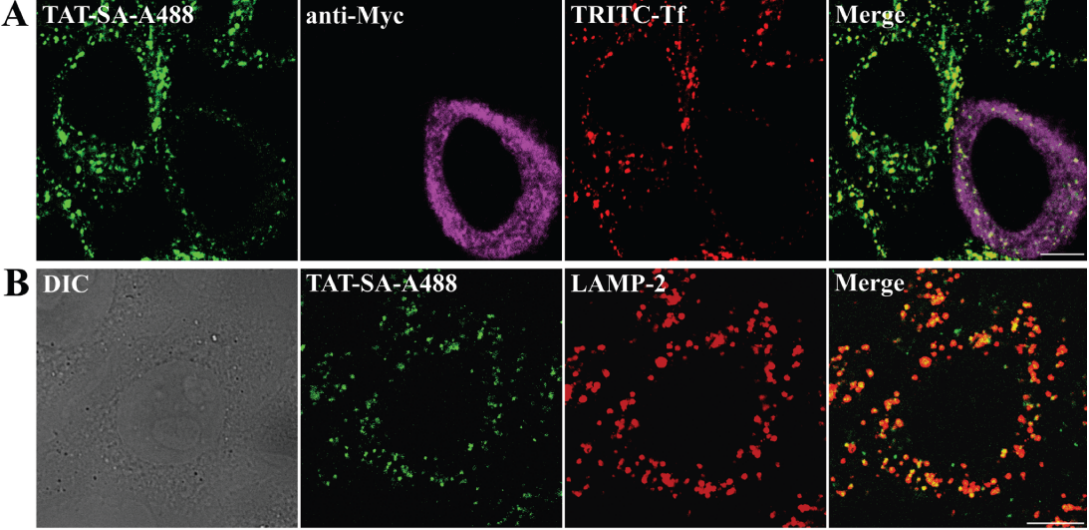
**Immunofluorescence microscopy studies of the cellular distribution of TAT-SA**. (A) HeLa cells were transfected with mutant AP180-C, a specific dominant-negative inhibitor of clathrin-mediated endocytosis, 24 h prior to transduction with TAT-SA-A488 (green) and clathrin-endocytosis marker TRITC-Transferrin (TRITC-Tf, red). Cells were fixed with PFA at 4 h post transduction, permeabilized with Triton-X and finally stained with antibody against myc-tagged AP180-C followed by an Alexa-633-conjugated anti-mouse antibody (purple). (B) The distribution of TAT-SA-A488 and lysosomal marker (red) was monitored in HeLa cells fixed (PFA) at 4 h post transduction and stained with an antibody against lysosomal marker LAMP-2 followed by an Alexa-546-conjugated anti-mouse antibody (red). Scale bars, 10 μm.

In order to characterize clathrin-dependent and clathrin-independent endocytosis in the uptake of TAT-SA, AP180-C plasmid encoding a dominant-negative form of AP180, an inhibitor of clathrin-mediated endocytosis, was used [28]. In HeLa cells overexpressing AP180-C, the internalization of TAT-SA was reduced, but not completely inhibited at 4 h post transduction (Fig. 3A), whereas the uptake of a clathrin-mediated endocytotic marker, Transferrin (Tf; 200 μg/ml), was markedly decreased. In the absence of AP180-C plasmid, immunofluorescence studies revealed slight colocalization of TAT-SA-A488 and an early endosomal marker (rab5) at 15 min post transduction as well as of TAT-SA-A488 and a recycling endosomal marker (rab11) at 30 min post transduction (unpublished data). In addition, the lysosomal marker LAMP-2 showed clear colocalization with TAT-SA-A488 at 2–4 h post transduction (Fig. 3B).

**Figure 4:**
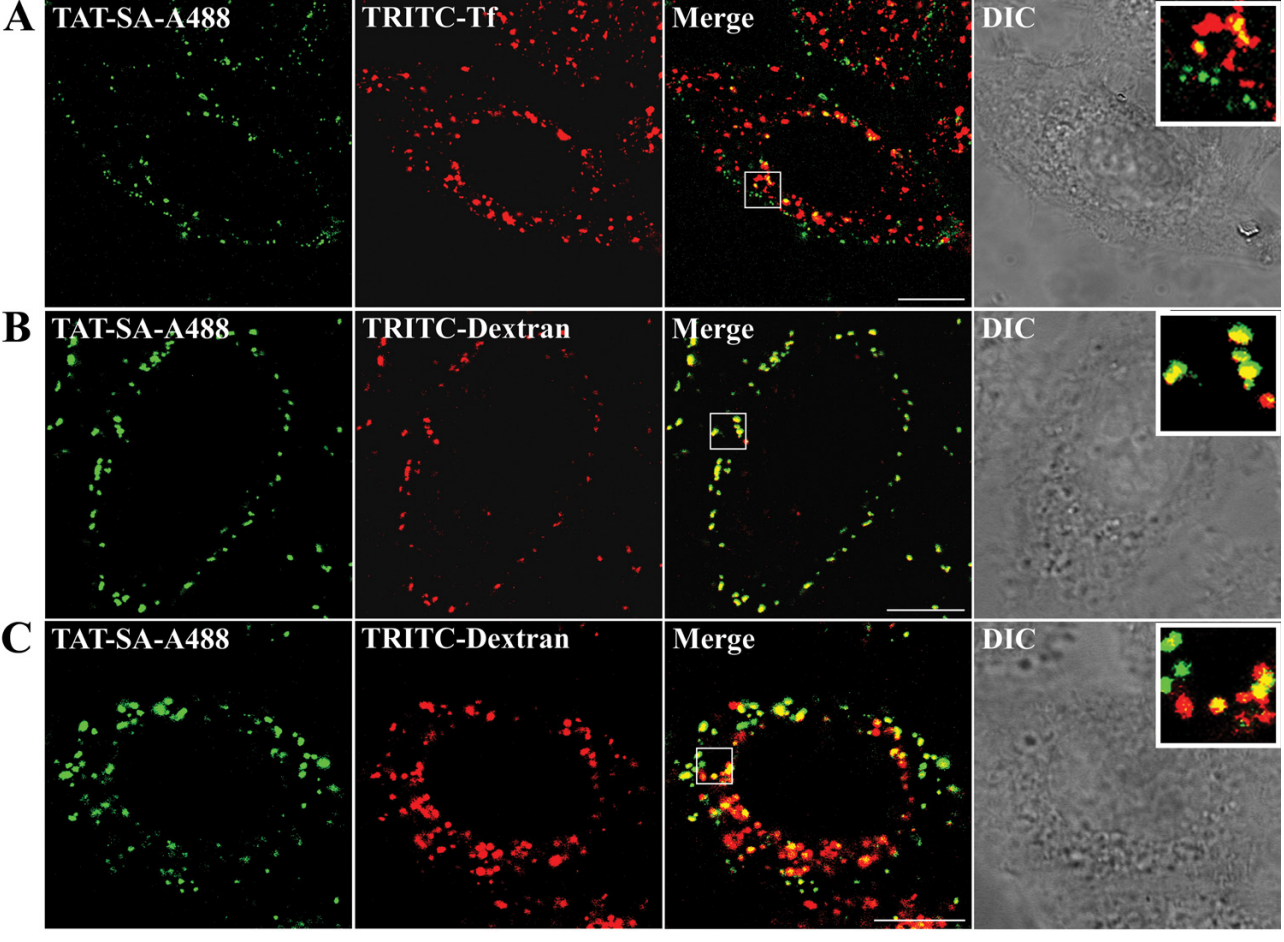
**Colocalization of TAT-SA with endocytic markers in living cells**. (A) HeLa cells were transduced first with TAT-SA-A488 (green) and then with TRITC-labeled transferrin (TRITC-Tf, red), a marker clathrin-mediated endocytosis, prior to analysis with confocal microscope at 15 min post transduction. (B) Cells transduced with TAT-SA-A488 and fluid-phase endosomal marker TRITC-labeled dextran (10 kD, red) were monitored at 15 min and (C) at 4 h post transduction. The white rectangles show the close-ups of representative structures. Scale bars, 10 μm.

**Figure 5:**
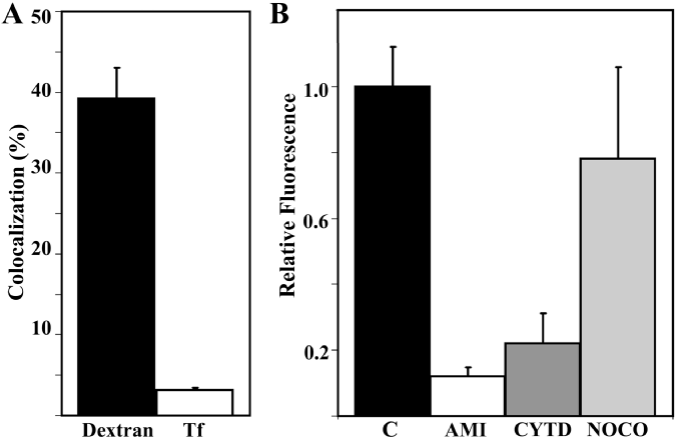
**Quantification of internalized TAT-SA in living HeLa cells**. (A) Quantitative analysis of the colocalization between both TAT-SA-A488 and TRITC-labeled transferrin and TRITC-labeled dextran (10 kD) at 15 min post transduction. (B) The relative fluorescence intensity of internalized TAT-SA-A488 was measured in cells treated with cytochalasin D (cytD), amiloride (ami) or nocodazole (noco) at 4 h post transduction. Control cells (C) were untreated. The fluorescence intensity data was collected from multiple series of cells by confocal microcopy and processed with the 3D LSM program.

The kinetics of TAT-SA translocation from the cell periphery towards the nuclear periphery at different times was monitored in living HeLa cells in the presence of the fluorescent endocytic markers transferrin and dextran. Partial colocalization of TAT-SA-A488 and TRITC-labeled Transferrin (TRITC-Tf) was observed first at 5 min post transduction in close proximity to the cell membrane and later at 15–30 min post transduction in perinuclear vesicles (Fig. 4A). In order to examine the role of non-clathrin-mediated endocytosis, a fluid-phase endosomal marker TRITC-labeled Dextran (TRITC-Dextran; 10 MW, 250 μg/ml), was used to monitor macropinocytic entry pathway. As shown in Figures 4B, 4C and in Additional File 2, extensive colocalization of TAT-SA-A488 and TRITC-Dextran was observed at both 15 min and 4 h post transduction. Moreover, quantitative analysis of the confocal images (n = 25–30) revealed an approximately 10-fold higher colocalization between TAT-SA-A488 and TRITC-Dextran (39.2% ± 3.8%) than TAT-SA-A488 and TRITC-Tf (3.1% ± 0.4%) at 15 min post transduction (Fig 5A).

The uptake of TAT-SA was further examined in living HeLa cells by quantitative image analysis (n = 25–30) in the presence of different endocytic inhibitors. In comparison to relative fluorescence intensity of untreated, transduced control cells (1.0 ± 0.3; Fig. 5B) a microtubule-disrupting drug, nocodazole, caused only a slight decrease (0.78 ± 0.3) of intracellular TAT-SA-A488. In cells treated with the filamentous F-actin elongation inhibitor cytochalasin D, affecting both macropinocytosis and clathrin-dependent endocytosis, a clear decrease (0.22 ± 0.1) in the amount of cytoplasmic TAT-SA was, however, measured. Furthermore, amiloride, an inhibitor of Na^+^/H^+ ^exchange required for macropinocytosis, displayed significantly reduced (0.14 ± 0.1) amounts of TAT-SA uptake. Importantly, in control experiments extensive disruption of microtubules or actin filaments was observed in cells treated either with nocodazole or cytochalasin D and amiloride. Additionally, cytochalasin D and amiloride markedly decreased the internalization of TRITC-Dextran in living cells (unpublished data).

### The endosomal release of TAT-SA and TAT-SA bound to biotin via the PPAA polymer

**Figure 6:**
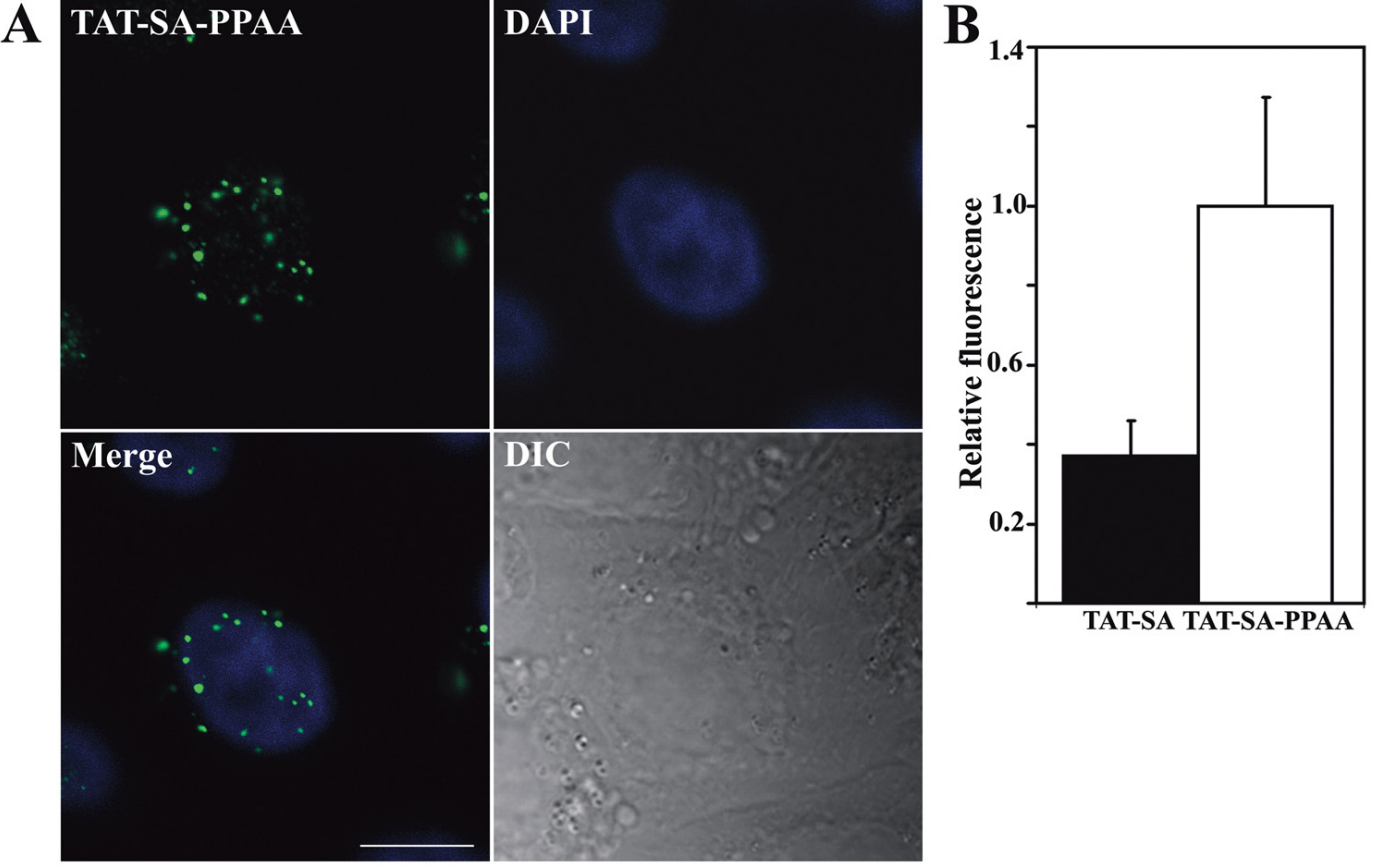
**Effect of PPAA polymer on nuclear import of TAT-SA in HeLa cells at 4 h post transduction**. (A) TAT-SA-A488 was first pretreated with biotinylated, endosomal-releasing peptide PPAA and then transduced into cells. Cells were fixed with PFA, permeabilized with Triton-X and immunolabeled with rabbit anti-SA Ab followed by Alexa-488-conjugated goat anti-rabbit IgG (green) and embedded with DAPI-containing Mowiol-Dabco (nucleus, blue). Scale bar, 10 μm. (B) Quantitative analysis of the relative nuclear fluorescence intensity of TAT-SA-488 in living cells in presence or absence of PPAA. Multiple series of optical sections of the nuclear area were collected by confocal microscopy prior to processing the data with the 3D LSM program.

The streptavidin-biotin association is one of the strongest known non-covalent interactions in nature. In order to alter the subcellular distribution of TAT-SA, a biotinylated, pH-responsive polymer poly(propylacrylic acid) (PPAA; 4 μM) was complexed to TAT-SA (2 μM) to promote endosomal release. The 3D illustrations of live HeLa cells showed that TAT-SA complexed with biotinylated PPAA was localized both in cytoplasmic vesicles and in the nucleus at 4 h post transduction (Fig. 6A, Additional file 3). Interestingly, TAT-SA was shown to accumulate into distinct nuclear foci, suggesting interactions with subnuclear components (Fig. 6A). Moreover, quantitative analysis (n = 25–30) of the relative nuclear fluorescence intensity demonstrated that PPAA induced over 2-fold increase in the intranuclear TAT-SA-488 (1.0 ± 0.3) when compared to TAT-SA-A488 transduced control cells (0.4 ± 0.1; Fig. 6B).

**Figure 7:**
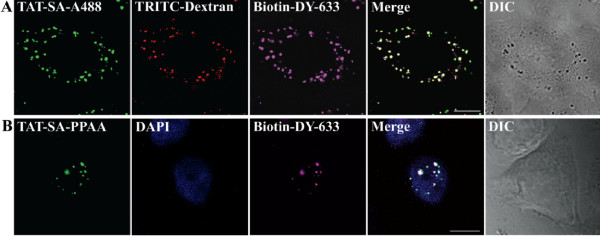
**Internalization and intracellular targeting of TAT-SA combined with biotin in HeLa cells**. (A) Cells were transduced with the complex of TAT-SA-A488 (green) and biotin-DY-633 (purple), followed by TRITC-labeled dextran (10 kD, red) prior to live cell imaging at 4 h post transduction (B). Cells were transfected with TAT-SA complexed with biotinylated PPAA and biotin-DY-633. Cells were fixed with PFA, permeabilized with Triton-X and immunolabeled with rabbit anti-SA Ab followed by Alexa-488-conjugated goat anti-rabbit IgG (green) and embedded with DAPI-containing Mowiol-Dabco (nucleus, blue). All images are 3D projections of the nuclear area. Scale bars 10 μm.

The ability of TAT-SA to act as an intracellular delivery vector by transporting biotin and biotinylated molecules into cells was also examined by confocal microscopy. As shown in the 3D illustration, TAT-SA-A488 (2 μM) and Biotin-DY-633 (1–2 μM) accumulated in large intracellular vesicles near the nucleus (Fig. 7A). No nuclear localization of TAT-SA was observed, however. Further experiments demonstrated strong colocalization of the TAT-SA-biotin complex and TRITC-Dextran in vesicles at the nuclear periphery at 2 h and 4 h post transduction, implying that most of the TAT-SA-biotin complexes were unable to escape from the intracellular vesicles. When endosomal releasing polymer PPAA was complexed with TAT-SA bound biotin, however, a major subnuclear accumulation was observed (Fig. 7B, Additional file 4).

Finally, cell viability experiments with MTT cell proliferation assay showed that TAT-SA (2 μM) was not cytotoxic to HeLa cells at 4–72 h post transduction (Table 1). Also no indication of a cytotoxic effect was observed in the presence of TAT-SA-complexed, biotinylated PPAA (4 μM) at 4–24 h post transduction and only a slight decrease in cell viability was detected at 48 and 72 h post transduction (94.0% ± 3.8% survival) compared to untransduced control cells (100.0% ± 3.3% survival).

**Table 1:** Cytotoxicity Assay

**p.t.**	**Control**	**TAT-SA**	**TAT-SA-PPAA**
24 h	100,0 ± 5,4	102, 5 ± 6,9	102,4 ± 1,6
48 h	100,0 ± 5,2	105,5 ± 5,7	96,4 ± 2,1
72 h	100,0 ± 3,3	99,0 ± 1,6	94,0 ± 3,8

MTT cytotoxicity assay of TAT-SA and TAT-SA-PPAA transduced HeLa cells at 24, 48 and 72 h post transduction. Survival percentages of three repetitive experiments and standard deviations are shown (control cells 100% survival).

## Discussion

The cell membrane is a barrier to the intracellular delivery of many pharmacologically important biological macromolecules. Several cell penetrating peptides have been shown to possess the ability to direct cellular uptake, including the HIV-1 TAT peptide. So far TAT has been used for directing the intracellular uptake of various biomolecular cargoes. In this study, however, a novel streptavidin fusion protein TAT-SA [10] was examined, which enables the transport of biotinylated cargoes into cells. The internalization of TAT-SA was characterized in both human cancer (HeLa and A549) and non-cancer cell lines (MRC-5) (Fig. 1A–E). The internalization of SA was a relatively slow process in which detectable amounts of SA-A488 were present in the cytoplasm but not the nucleus of HeLa cells at 4 h post transduction (Fig. 1G–H). On the contrary, TAT-SA displayed rapid and efficient internalization in living HeLa cells starting at 5 min post transduction with distribution into vesicular structures in the cytoplasm after 15 min (Fig. 1A, 1D–E). At later time points, confocal and EM imaging demonstrated that the majority of TAT-SA was localized in the cytoplasmic vesicles with trace amounts in the nucleus (Fig, 1D–E, Fig. 2A and 2B). Previously, numerous possible internalization routes for TAT have been proposed, such as lipid-raft-mediated macropinocytosis [21], caveolae-mediated endocytosis [19,20] and clathrin-independent and dependent endocytosis [22,23]. However, the uptake characteristics of the TAT peptide alone and of TAT-conjugated cargoes have been demonstrated to differ significantly [1,4,22]. Furthermore, TAT-mediated internalization process has proposed to be dependent on the properties of the cargo molecule, TAT concentration and cell line [1,29,30]. In our study, streptavidin (60 kD) as a larger partner of the TAT-SA fusion construct (TAT_47–57_-peptide, 11aa) is likely to affect the uptake and intracellular trafficking of the vector. Notably, we show here that direct microinjection of high concentrations of TAT-SA or SA into the cytoplasm resulted in efficient nuclear uptake of TAT-SA but not SA (Fig. 1F and 1I). This verifies previous findings that upon introduction into the cytosol, the TAT peptide is capable of mediating the nuclear import of its streptavidin fusion partner. Moreover, it is known that positively charged molecules internalize the cells efficiently. Consequently, taken to account that the plain SA is negatively charged (theoretical pI 6.04) and TAT-SA is positively charged (theoretical pI 9.92), TAT-SA internalizes the cells more efficiently than SA. Taken together, these data demonstrate that TAT-SA is efficiently internalized into various human cells but that only a relatively small proportion is further released into the cytoplasm and transported into the nucleus, most likely reflecting the inability of TAT-SA to escape from endocytic vesicles.

In order to use TAT-SA as a vector to deliver biotinylated molecules into cells, the internalization and delivery mechanisms of the construct have to be characterized *in vitro *and *in vivo*. In recent years a number of studies have suggested a variety of internalization routes for TAT peptide and TAT-mediated cargoes. To study this, three major endocytic pathways involving caveolae, lipid rafts and clathrin-coated pits were analyzed using specific endocytic markers, inhibition agents and immunofluorescence labelings of each pathway. Previous studies have suggested the TAT peptide enters cells by temperature-dependent, caveolae-mediated endocytosis [19,20]. However, no evidence for the use of the caveolae route in the internalization of TAT-SA was observed in the present confocal microscopial colocalization studies with a caveolar marker protein. Moreover, treatment with methyl-β-cyclodextrin, a cholesterol depletion agent known to inhibit the caveolae route [31] or transduction of the caveolin-deficient HepG2 (unpublished data) and Jurkat T cells [10,21,32,33] did not prevent the entry of TAT-SA-A488 into living cells. Altogether, these data imply that endosomal routes other than caveolae-mediated entry are required for the uptake of TAT-SA.

It has been suggested that the internalization of SA occurs via clathrin-mediated endocytosis [24,25]. Here, the role of clathrin-dependent uptake of TAT-SA was monitored in cells overexpressing the mutant AP180-C protein [34]. AP-180C is required for the efficient assembly of clathrin-coated pits by interacting with the clathrin heavy chain through its C-terminal clathrin-binding motifs. Our data indicated that in AP180 overexpressing cells the internalization of TAT-SA was only slightly affected, whereas the uptake of Tf, a marker of clathrin-dependent endocytosis [35], markedly decreased (Fig. 3A). The double-immunolabeling studies of clathrin-mediated endocytosis showed only minor colocalization of TAT-SA with early and recycling endosomal markers at early time points (unpublished data). However, at later stages of endocytosis TAT-SA accumulated in lysosomes, the final destination of multiple endocytic pathways (Fig. 3B). Clathrin-mediated endocytosis seemed therefore to be only partially involved in the uptake of TAT-SA, the major internalization being mediated by another, more efficient pathway.

**Figure 8:**
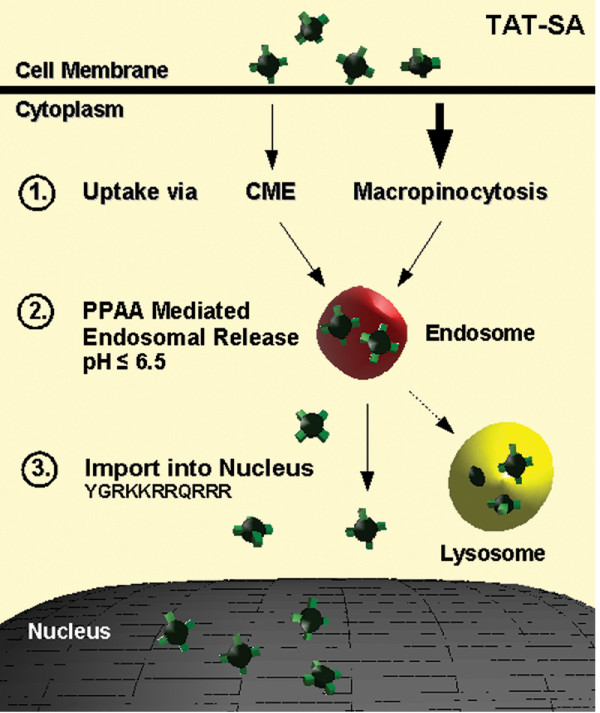
**Schematic model illustrating internalization of TAT-SA-PPAA in the human cell**. After interaction with the cell surface, TAT-SA enters the cell via clathrin-mediated endocytosis or macropinocytosis (1), followed by transport to endosomal vesicles. Later, at low pH (≤6.5), PPAA induces the escape of TAT-SA from endosomes (2) and the nuclear localization sequence of the TAT peptides mediates the nuclear import of the construct (3). In the absence of PPAA polymer most of TAT-SA remains trapped in endosomes and is transported and further degraded in lysosomes.

Many oligoarginine peptides have been proposed as candidates for cellular internalization via macropinocytosis [30]. Recently, uptake via clathrin-independent endocytosis was also suggested in the internalization of TAT-Cre and biotinylated TAT [21,23,36]. In our study, the intracellular localization of TAT-SA was monitored in living HeLa cells with various fluorescent endocytic markers. Shortly after internalization, only slight colocalization was detected with TAT-SA and Tf, a clathrin-dependent endocytic marker (Fig. 4A), whereas extensive colocalization between TAT-SA and the fluid-phase endosomal and macropinocytic marker dextran was observed (4B, 4C and Additional file 2). Quantitative measurements of fluorescent intensity at 4 h post transduction displayed an approximately 10-fold increase in the colocalization of TAT-SA and dextran as compared to TAT-SA and Tf (Fig. 5A), thus supporting previous observations of the role of macropinocytosis as an entry mechanism for TAT. Additionally, when cells were treated with amiloride, a known inhibitor of macropinocytosis [37], the uptake of TAT-SA-A488 was almost entirely inhibited. In the presence of cytochalasin D, an F-actin-disrupting agent affecting both macropinocytosis and clathrin-mediated endocytosis [34,38], only a minor internalization of TAT-SA-A488 occurred (Fig. 5B). To conclude, we were able to establish macropinocytosis as a major internalization pathway of TAT-SA, as illustrated (Fig. 8).

The central interest in this study was to determine the ability of TAT-SA to act as a carrier of biotinylated molecules into nucleus of human cells. Although numerous studies have been conducted on TAT-related protein therapeutics, only a few have attempted to address the intracellular release of macromolecules from endocytic vesicles. Various approaches for endosomal escape have been proposed, such as endosome-disrupting agents (HA2, 4_3_E), acid-sensitive linkers or laser illumination [39]. In a recent study by Wadia *et al*. (2004) it was shown that an internalized TAT-Cre fusion protein was capable of nuclear uptake and the subsequent induction of reporter gene expression. Most of the TAT-Cre peptides were, however, observed to reside in cytoplasmic vesicles and therefore the endosomal escape was enhanced by incorporation of the HA2 peptide from the influenza virus hemagglutin protein. Albarran *et al*. (2005) have previously shown the delivery of biologically active alkaline phosphatase (140 kD) and R-phycoerythrin (240 kD) into the cytoplasm of Jurkat T cells by TAT-SA. In present study, it was, however, shown that both TAT-SA and TAT-SA bound to biotin remained enclosed in endocytic vesicles for several hours after internalization (Fig. 4C, 7A, Additional file 3). Therefore, the effect of a biotinylated pH-responsive polymer PPAA [10] on the subcellular distribution of TAT-SA was investigated. In previous studies PPAA has shown to disrupt endosomes at pH 6.5 or below, causing the cytosolic release of cargo molecules [10,40-43]. Recently, PPAA has also been shown to enhance the delivery of antibody-targeted conjugates into the cytoplasm [40] and PPAA-containing lipoplexes have improved wound healing in mice [42]. Our data indicated clearly that the nuclear import of TAT-SA-A488 or TAT-SA bound to biotin was considerably increased when combined with biotinylated PPAA (Fig. 6A and 7B, Additional file 4). Quantitative analysis of live cells showed an approximately 2-fold increase in the nuclear localization of TAT-SA-A488-PPAA complexes compared to TAT-SA-A488 alone (Fig. 6B). The endosomal escape of TAT-SA-A488-PPAA may, however, been more extensive, since the detection of fluorescence intensity by confocal microscope is not sensitive enough to observe all the nuclear fusion proteins. Importantly, no significant cytotoxicity of TAT-SA-PPAA was observed by MTT assay even at 72 h post transduction (Table 1). Overall, these data further demonstrate that the incorporation of PPAA with TAT-SA alters the subcellular distribution of the internalized fusion protein, resulting in improved nuclear delivery of its biotin partner (Fig. 8).

Recently, several therapeutic applications for TAT-mediated cellular internalization have been developed, including the transport of an inhibitor for human papillomavirus type 16 [44] and for the apoptosis-promoting caspase-3 protein used in HIV-therapy [45], the extension of the cytotoxic activity of herpes simplex virus-1 thymidine kinase for cancer therapy [46], dendritic cell-based immunotherapy [47] and enhancement of viral-mediated gene delivery, [48] to name just a few. In this study, the fusion of TAT with SA imparts the versatility and precision of the SA-biotin system and allows the complexation of numerous organic and inorganic cargoes. Additionally, the TAT-SA fusion protein and its use in combination with the endosomal-releasing polymer PPAA demonstrates the potential of this construct for delivering biotinylated molecules into various intracellular compartments, depending on the chemistry of the chosen biotin partner.

## Conclusion

Non-viral vector TAT-SA internalizes into human cells via both macropinocytosis and clathrin-dependent endocytosis. The subcellular distribution of TAT-SA is significantly altered through the incorporation of a pH-sensitive polymer PPAA. Here, we have characterized of a novel and versatile vector that is capable of delivering an array of biotinylated macromolecular cargoes into cells.

## Methods

### Cells

HeLa, A549, MRC-5 and HepG2 cell lines were obtained from the American Type Culture Collection (ATCC, Manassas, VA). They were grown in monolayer cultures in Dulbecco's modified Eagle's medium (DMEM, MEM), supplemented with 10% inactivated fetal calf serum and penicillin-streptomycin (Gibco BRL, Paisley, UK) at 37°C, in 5% CO_2_. For HepG2 cells non-essential amino acids (Gibco BRL) and Na-pyruvate (Merck & Co. Inc., Whitehouse Station, NJ) were also used.

### Antibodies and other reagents

The rabbit polyclonal antibody against streptavidin was the generous gift from Edward Bayer (The Weizmann Institute of Science, Rehovot, Israel). The following polyclonal antibodies (Ab) and monoclonal antibodies (MAb) were used to detect endocytic vesicles and cytoskeleton: rab5 MAb (Transduction Laboratories, Lexington, KY); caveolin-1 MAb and Rab11 Ab (Zymed Laboratories, South San Fransisco, CA); LAMP-2 MAb (Biotechnology Associations Inc. Birmingham, AL); tubulin MAb and actin Ab (Sigma Aldrich, St Louis, MO). The myc MAb (9E10) was obtained from the ATCC. Anti-rabbit IgG AP-conjugate was from Promega (Madison, WI). In the double- and triple-immunolabeling studies Alexa-546- or Alexa-633-conjugated anti-mouse antibodies and Alexa-633-conjugated anti-rabbit antibodies were from Molecular Probes (Eugene, OR). Nanogold-conjugated polyclonal rabbit immunoglobulin G (IgG) was purchased from Nanoprobes (Yaphank, NY).

Streptavidin (SA), nocodazole, cytochalasin D, methyl-β-cyclodextrin, amiloride and biotin were from Sigma. TRITC-Dextran (10.000 MW) and TRITC-Tf were from Molecular Probes. Biotin-DY-633 was obtained from Dyomics GmbH (Jena, Germany). Biotinylated-PPAA (poly(propyl-acrylic acid); 11 kD) was prepared as previously described [10,40,41,49]. Nanogold and HQ-silver enhancement reagents were obtained from Nanoprobes. Epon LX-112 was purchased from Ladd Research industries (Williston, VT).

### TAT-Streptavidin (TAT-SA) construction and expression

The design and construction of the TAT-SA gene, T7 expression system (pET-21a, Novagen, Inc., Madison, WI) and the isolation, refolding, purification as well as structural and preliminary functional characterization of the TAT-SA fusion protein have previously been reported [10]. TAT-SA and SA was labeled with Alexa-488 according to the protocol for amine-reactive probes (Molecular Probes). The biotin-binding ability and stability of protein constructs were detected by SDS-PAGE and immunoblot analysis. In these experiments part of the samples were first conjugated with biotin at RT for 15 min, and then TAT-SA and biotinylated TAT-SA samples were preheated to 22°C, 37°C or 68°C for 10 min. All samples were predisposed to a reducing agent β-mercaptoethanol for 30 min prior to loading.

### Transduction of TAT-SA proteins

For the internalization studies cells were grown to subconfluency on coverslips and entry of TAT-SA (2 μM) was monitored in Hela, A549, MRC-5 and HepG2 cells fixed at 4 h post transduction. Moreover, intracellular localization of TAT-SA-A488 (2 μM) and SA-A488 (2 μM) was analyzed in living HeLa cells at 4 h post transduction. For cointernalization of TAT-SA with various fluorescent endocytic markers, HeLa cells were first transduced with TAT-SA-A488 for 5 min, then fed with TRITC-labeled Transferrin (TRITC-Tf, 200 μg/ml) or TRITC-labeled Dextran (250 μg/ml) and finally monitored at different times by confocal microscopy (Zeiss LSM 510 coupled to a Zeiss Axiovert 100 M, Karl Zeiss, Jena, Germany). All live cell imaging of the internalization process was monitored in 0.75-μm confocal sections, and summarized as 3 focal plane images.

In the experiments with different endocytic inhibitors HeLa cells were preincubated in medium containing methyl-β-cyclodextrin (2.5 mM), nocodazole (60 μM), cytochalasin D (4 μM) or amiloride (0.4 mM) for 30 min, followed by TAT-SA-A488 transduction. All treatments were maintained up to and including monitoring in living cells. In control studies, the cells were fixed and depolymerization of microtubules or actin was verified by immunolabeling of tubulin or actin.

To examine the intracellular distribution of TAT-SA with biotin and the effect of the endosomal releasing polymer PPAA, live cell studies were performed with TAT-SA and fluorescent biotin and/or biotinylated-PPAA. TAT-SA-A488 (2 μM) was incubated with Biotin-DY-633 (1–2 μM) and/or biotinylated-PPAA (4 μM) at room temperature (RT, 20–23°C) for 15–30 min, transduced into cells at 37°C for 4 h and monitored in living or fixed HeLa cells.

For live cell studies by a laser scanning confocal microscope (Zeiss LSM 510) the cells were maintained in Scotch chambers [50] in CO_2_-independent medium (Gibco) supplemented with 10% inactivated FCS and penicillin-streptomycin. Prior to detection the objective and sample holder were heated to 37°C. In the imaging, appropriate excitation and emission settings together with the multitracking mode were used to avoid false colocalization.

To quantitate the colocalization of TAT-SA-A488 with TRITC-Tf or TRITC-Dextran, the collected intracellular fluorescence intensity data for each cellular fluorescent marker was processed using 3D LSM and ImageJ programs (Colocalization Finder Plug-in). The influence of different drugs on the cellular internalization of TAT-SA-488 was quantitated by collecting the intracellular fluorescence intensity data from multiple series of drug-treated cells and then processed with the 3D LSM program. Quantitative analysis corresponding to the nuclear import of TAT-SA-488 in the presence of PPAA was performed by summarizing the fluorescence intensity data from multiple series of optical sections of the nuclear area and analyzing with the 3D LSM program. Prior to all the quantitative analyses the data from each channel were corrected by reducing the background signal of untransduced cells.

For immunofluorescence microscopy, the cells were fixed at set time intervals post transduction either with absolute methanol (-20°C) at RT for 6 min or with 4% paraformaldehyde (PFA) in phosphate buffered saline (PBS, pH 7.4) at RT for 20 min. PFA fixed cells were permeabilized with 0.5% Triton X-100. Cells were sequentially immunolabeled with primary and labeled secondary antibodies, embedded with Mowiol-DABCO or ProLong^® ^Gold antifade reagent with DAPI (Molecular Probes) and subjected to confocal microscopy (Zeiss LSM 510 or Olympus Fluo-View 1000, Olympus Optical Co., Tokyo, Japan).

### Microinjection

Hela cells were grown to subconfluency on microgrid coverslips (grid size, 175 nm; Eppendorf, Hamburg, Germany). The injections were performed into the cytoplasm of living cells using a semiautomatic system comprising a Transjector 5246 and Micromanipulator 5171 (Eppendorf) attached to an inverted microscope. Capillaries for injections (Clark Electromedical Instruments, Pangbourne, UK) were prepared with a model P97 capillary puller from Sutter Instruments (Novato, CA). The sample ejection volume (0.1 pl) was measured by injecting radioactive [^3^H]-biotin (Amersham Biosciences, Little Chalfont, UK) standard. Since the concentration of injected TAT-SA-A488 and SA-A488 was 1.4 mg/ml in PBS, the actual intracellular amount of the injected proteins was estimated to be approximately 1.5 × 10^6 ^molecules/cell.

### Nanogold pre-embedding immunoelectron microscopy

After transduction of TAT-SA (5 μM) and fixing with glutaraldehyde, HeLa cells were washed with phosphate buffer (0.1 M Na_2_HPO_4 _pH 7.4) and permeabilized with a saponin buffer (0.01% saponin/0.1% BSA/0.1 M Na_2_HPO_4_). Cells were then labeled with anti-SA Ab at RT for 1 h, followed by nanogold-conjugated anti-rabbit IgG at RT for 1 h. After appropriate washes with saponin and phosphate buffers, post-fixing (1% glutaraldehyde in 0.1 M phosphate buffer) and quenching (50 mM NH_4_CL in Na_2_HPO_4_) were performed. Moreover, silver enhancement and gold toning (2% Na-acetate, 0.05% HAuCl_4_, and 0.3% Na-thiosulphate in EM water) were followed by post-fixing (1% osmium tetroxide, 0.1 M phosphate buffer, K_4_Fe(CN)_6 _15 mg/ml) at 4°C for 1 h. The samples were dehydrated in ethanol, stained with 2% uranylacetate, and embedded in LX-112 epon. Polymerization of epon was performed over a 24 h period, first at 45°C and then at 60°C, after which the samples were stained with toluidine blue, cut with an ultramicrotom (Reichert-Jung, Ultracut E) and stained again with uranylacetate and lead citrate. Detection was performed by a JEOL JEM-1200EX transmission electron microscope operated at ~60 kV.

### Overexpression of the specific dominant-negative inhibitor of clathrin-mediated endocytosis

The AP180-C mutant construct was the generous gift of Dieter Blaas (University of Vienna, Austria). Following the manufacturer's protocol, HeLa cells were grown to 50% confluency on coverslips and transfected by FuGENE 6 reagent (Roche, Basel, Switzerland) with Qiagen-purified (Santa Clarita, CA) plasmid (2.5 μg/7 cm^2 ^dish), encoding for the myc-tagged assembly protein 180 (AP180-C). Two days after transfection the cells were transduced with TAT-SA-488 for 4 h at 37°C, fixed and immunolabeled with the myc MAb as described above.

### Cytotoxicity Assay

Cytotoxicity of TAT-SA (2 μM) and TAT-SA-PPAA (4 μM) complexes were determined by CellTiter 96^® ^Aqueous One Solution Cell Proliferation Assay (MTT assay; Promega) according to the manufacture's protocol. The measurements were performed by a spectrophotometer (Wallac Victor^2 ^1420 Multilabel Counter and Wallac Workout™ data management software; Perkin Elmer Life Sciences, Boston, MA) at an absorbance of 492 nm. Viability of cells was calculated by comparison of the absorbance in control cells (100% survival) and TAT-SA or TAT-SA-PPAA treated cells.

## Authors' contributions

JR designed the study, performed most of the experiments and drafted the manuscript. BA and PSS performed vector construction [10]. JJ, TOI, PK and VPH assisted in imaging and image analysis. MSK, BA and PSS participated in the design and execution of this manuscript. MV managed the project and had input at all stages of the study. All authors read and approved the final manuscript.

## Supplementary Material

Additional file 1 **Characterization of TAT-SA constructs by SDS-PAGE and Western Blot Analysis**. TAT-SA (lanes 1, 3) and TAT-SA bound to biotin (lanes 2, 4) are shown as tetrameric conformations (60 kD) in reducing conditions mimicking the cellular environment at 22°C or after preheatment to 37°C. Click here for file

Additional File 2 **The role of macropinocytosis in the internalization of TAT-SA in living HeLa cells**. Three-dimensional (3D) illustration of colocalization of TAT-SA-A488 (green) and macropinocytosis marker TRITC-labeled dextran (10 kD, red) at 15 min post transduction by confocal microscopy. Merged 3D images of TAT-SA-A488 and TRITC-dextran together (yellow) with DIC images were used for construction of the QuickTime movie. Click here for file

Additional file 3 **The cytoplasmic distribution of TAT-SA-biotin**. Three-dimensional (3D) illustration of localization of TAT-SA-A488 (green) complexed with Biotin-DY-633 (purple) in the living cells at 4 h post transduction (colocalization, white) by confocal microscopy. The merged 3D images were used to construct the QuickTime movie. The close-up box shows the magnified 3D projection slice of the nuclear area. Click here for file

Additional file 4 **The cytoplasmic and nuclear distribution of TAT-SA in the presence of biotinylated PPAA polymer**. Three-dimensional (3D) illustration of localization of TAT-SA-A488 (green) complexed with biotinylated endosomal-releasing peptide PPAA in the living cells at 4 h post transduction by confocal microscopy. The merged 3D images of the different structures were used to construct the QuickTime movie. The close-up box shows the magnified 3D projection slice of the nuclear area. Click here for file
