# Supplementary figures and images for: Internalization of novel non-viral vector TAT-streptavidin into human cells
Source: BMC Biotechnol. 2007 Jan 2;7:1. doi: 10.1186/1472-6750-7-1 (PMC1779780; doi:10.1186/1472-6750-7-1)

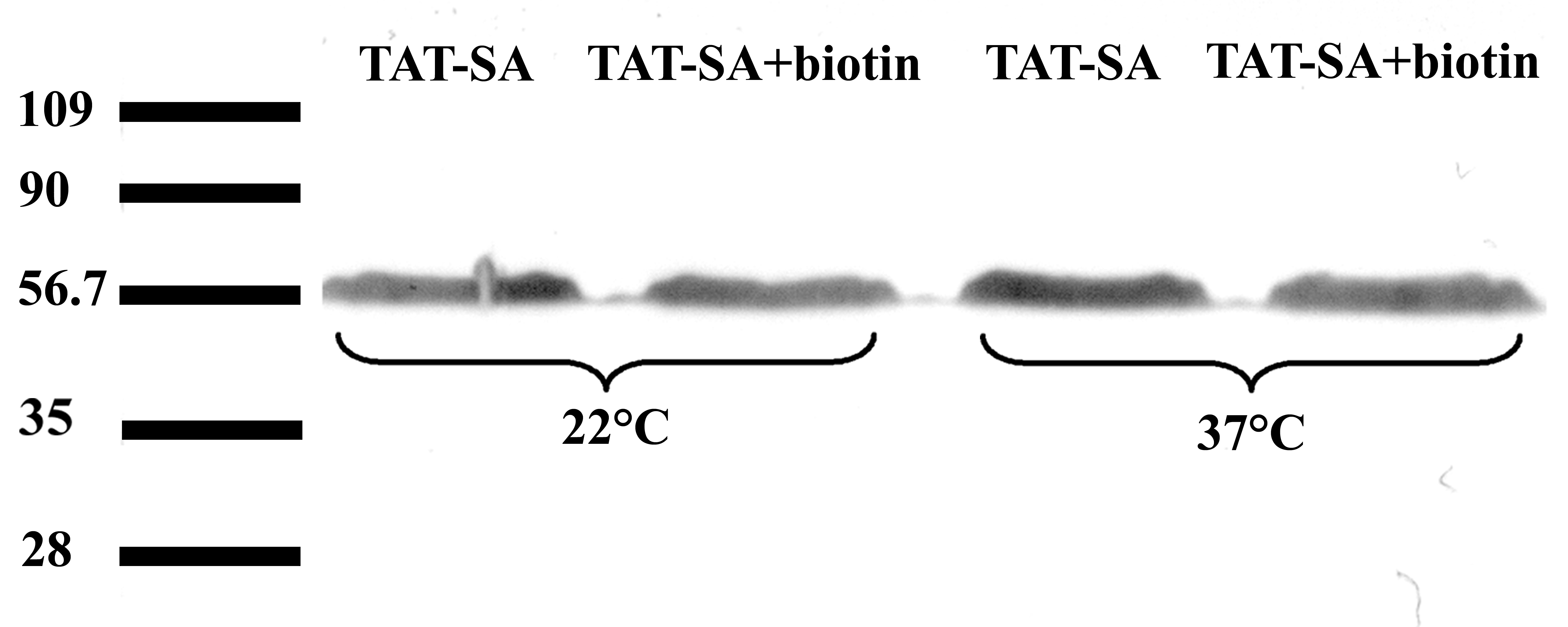

Supplement: Additional file 1 — Characterization of TAT-SA constructs by SDS-PAGE and Western Blot Analysis. TAT-SA (lanes 1, 3) and TAT-SA bound to biotin (lanes 2, 4) are shown as tetrameric conformations (60 kD) in reducing conditions mimicking the cellular environment at 22°C or after preheatment to 37°C. [file 1472-6750-7-1-S1.tiff]
